# Natural Deep Eutectic Solvent-Based Extraction of Flavonoids from *Citrus Aurantium* L. By-Products: Process Optimization and Physicochemical Characterization of Optimal System

**DOI:** 10.3390/molecules31183192

**Published:** 2026-09-10

**Authors:** Joaquín Fernández-Cabal, Kevin Alejandro Avilés-Betanzos, Manuel Octavio Ramírez-Sucre, Juan Valerio Cauich-Rodríguez, Ingrid Mayanin Rodríguez-Buenfil

**Affiliations:** 1 Centro de Investigación y Asistencia en Tecnología y Diseño del Estado de Jalisco A.C., Subsede Sureste, Tablaje Catastral 31264, Km. 5.5 Carretera Sierra Papacal-Chuburná Puerto, Parque Científico Tecnológico de Yucatán, Mérida 97302, Yucatán, Mexico; jofernandez_al@ciatej.edu.mx (J.F.-C.); keaviles_al@ciatej.edu.mx (K.A.A.-B.); oramirez@ciatej.mx (M.O.R.-S.); 2 Centro de Investigación Científica de Yucatán, Unidad de Materiales, Calle 43 No. 130 x 32 y 34, Colonia Chuburná de Hidalgo, Mérida 97205, Yucatán, Mexico

**Keywords:** *Citrus aurantium* by-products, central composite design, total flavonoid content, response surface methodology, NADES

## Abstract

*Citrus aurantium* L. by-products are a valuable source of bioactive compounds with potential industrial applications. This study evaluated the use of natural deep eutectic solvents (NADESs), formulated with choline chloride as the hydrogen bond acceptor (HBA) and fructose as the hydrogen bond donor (HBD), combined with ultrasound-assisted extraction to optimize the recovery of flavonoids from *Citrus aurantium* L. industrial by-products. A central composite design (CCD) was employed to optimize total flavonoid content (TFC). The molar ratio (MR; 1 and 2 mol of HBD per mol of HBA) and the percentage of added water (AW; 40 and 60%) were optimized. In addition to TFC, total phenolic content (TPC), total ascorbic acid (TAA), antioxidant capacity (Ax) and phenolic profiles were also evaluated. The optimal extraction conditions, as obtained by Response Surface Methodology (RSM) for TFC, corresponded to an HBD molar ratio of 1.33 per 1 mol of HBA and 47.48% added water, resulting in an optimal value of 1576.63 ± 4.9 mg quercetin equivalents (QE)/100 g dry mass (DM). Maximum values of 4276.94 ± 25.4 mg gallic acid equivalents (GAE)/100 g DM for TPC, 2077.66 ± 21.3 mg/100 g DM for TAA and 88.09 ± 0.1% inhibition for Ax were also obtained. The optimal NADES was characterized by density, viscosity, pH, Fourier-transform infrared (FTIR) and Raman spectroscopy. Additionally, phenolic profiling revealed high concentrations of hesperidin (2747.1 mg/100 g DM), naringenin (1251.93 mg/100 g DM), and quercetin + luteolin (660.65 mg/100 g DM).

## 1. Introduction

In recent years, growing concern about the environmental impact of human activities has changed consumer preferences, with increasing interest in goods and services developed under sustainability criteria. This trend has encouraged industry to adopt and increase environmentally friendly practices, promoting the development of natural products and reducing the use of synthetic compounds that may be harmful to ecosystems. In particular, the production of foods obtained through sustainable practices has gained increasing attention due to growing concerns about the conservation of natural resources, biodiversity, and public health. Consequently, there is growing interest in developing technologies that enable the efficient and environmentally friendly extraction of biomolecules, such as proteins, lipids, carbohydrates, vitamins and bioactive compounds including polyphenols [1,2].

Polyphenols are secondary metabolites synthesized by plants in response to different stress conditions. They are classified into five major groups, flavonoids, phenolic acids, stilbenes, lignans, and tannins, which are further subdivided according to the structure of their basic skeleton; among these, flavonoids have been the most extensively studied due to their wide range of biological activities and pharmacological effects, including immunomodulatory, hypoglycemic and antibacterial activities, as well as their role in the prevention of numerous diseases, such as cancer, Alzheimer’s disease, and diabetes, among others. Flavonoids can be found in plants, fruits, and vegetables, making plant-derived materials an important source of these compounds [3,4].

Every year, more than 190 million tons of agro-industrial by-products are generated. These are often disposed of on landfills or other waste disposal sites, where their slow and progressive degradation contributes to greenhouse gas emissions, as well as air, water, and soil pollution, while also occupying vast areas that could otherwise be used for agriculture. Among the main sources of these by-products are the processing of oilseed crops, vegetables, and horticultural crops, as well as residues obtained from plants such as grapes, pomegranates, melons, and, in particular, citrus fruits [5,6].

Citrus fruits belong to the Rutaceae family and, due to their attractive colors, aromas, and flavors, are one of the most popular fruits worldwide. Representative citrus species around the world are mandarin (*Citrus reticulata* Blanco), sweet orange (*Citrus sinensis* L.), lemon (*Citrus limon* L.), grapefruit (*Citrus paradisi* L.) and lime (*Citrus aurantifolia* L.) [7]. In 2021, citrus fruits ranked as the second most produced fruit crop worldwide (after bananas and plantains), with a total production of 161.8 million tons cultivated on more than 10.2 million hectares. Among citrus fruits, oranges were the most important crop, with a production of 75.57 million tons, accounting for 46.7% of the total, cultivated at 9.93 million hectares. Mandarins ranked second, with a production of 41.95 million tons (25.9% of total citrus production) grown on 3.11 million hectares. Finally, limes and lemons reached a combined production of 20.83 million tons, representing 12.87% of total citrus production, cultivated on 1.34 million hectares, with China, Brazil, India, the United States and Mexico ranking among the top 10 producers of citrus [8].

*Citrus aurantium* L., commonly known as bitter or sour orange, is a hybrid of *Citrus maxima* (pomelo) and *Citrus reticulata* (mandarin) [9]. It is an evergreen tree characterized by its small, fragrant white flowers. It can reach up to 5 m in height and is believed to have originated in Eastern Africa and southeast Asia, specifically in Syria. It can be found in several varieties, including *amara*, *dulcis*, and *sinensis*, among others. Although it is not among the most widely produced citrus varieties, it has a great importance in traditional Chinese medicine, where it is produced and consumed in large quantities [10,11]. However, *C. aurantium* L. is also used in other regions of the world, such as the Yucatán Peninsula, México, where its gastronomic importance has made it one of the main ingredients in the preparation of traditional dishes. Its importance is such that processing plants in the region currently extract its juice and essential oils for export, generating considerable amounts of by-products mainly composed of flavedo, albedo, pulp, and seeds [12].

In this context, green chemistry, based on 12 fundamental principles that seek to prevent pollution through the optimization of product and process design, with the aim of reducing or, ideally, eliminating the use and generation of hazardous substances [13,14], has among its objectives the valorization of industrial by-products, as well as the design and implementation of extraction processes that reduce energy consumption, employ alternative solvents, generate renewable natural products, and ensure that the resulting extracts or products are safe and of high quality [15]. Green extraction offers several advantages, including higher extraction efficiency, lower solvent consumption, and the preservation of valuable bioactive compounds, overcoming the limitations of conventional methods, such as low extraction yields, high solvent consumption and the risk of degradation of thermosensitive compounds like flavonoids [16].

Natural deep eutectic solvents (NADESs) are a representative example of the implementation of green chemistry. They are solvents formed by mixing two or more pure compounds in a specific ratio to create a eutectic system with non-ideal thermodynamic behavior. This behavior results from the strong interactions of the hydrogen bonds and van der Waals forces between the components, where one component acts as a hydrogen bond donor (HBD) and the other as a hydrogen bond acceptor (HBA), leading to a decrease in the melting point due to intermolecular interactions [17]. NADESs are considered natural because they are composed of primary or secondary metabolites. These metabolites, which plants, bacteria, fungi, and animals use for their physiological processes and survival, include sugars, organic acids, organic bases, and amino acids [18]. Their extraction efficiency of bioactive compounds is determined by the conditions used during their preparation, where factors such as the HBA and HBD selection, molar ratio, and water addition play an important role, as they provide important physicochemical properties related to their extraction capacity, such as polarity, viscosity, density and pH [19,20]. Therefore, statistical optimization approaches can be applied to determine the optimal extraction conditions.

Response surface methodology (RSM) is a mathematical and statistical technique for the design of experiments that aims to optimize a response influenced by several independent variables by describing the relationship between the factors and the response [21,22]. These models are used to evaluate the influence of the factors and their interactions on the response, as well as to optimize the process. Statistical optimization experiments can be performed using different experimental designs, which differ in the selection of experimental points and the number of experimental runs.

Therefore, the aim of this study was to optimize the formulation of a fructose-based NADES using choline chloride as the HBA and ultrasound-assisted extraction to maximize the recovery of flavonoids from *Citrus aurantium* L. by-products. In addition to the optimization of total flavonoid content (TFC), the extracts obtained under each experimental condition were evaluated for their total polyphenol content (TPC), total ascorbic acid (TAA), antioxidant capacity (Ax), and individual phenolic profile. Finally, the optimal NADES was physicochemically characterized through viscosity, density, pH, Raman and Fourier Transform Infrared Spectroscopy (FTIR) analyses to provide a comprehensive understanding of the solvent system associated with the highest extraction performance.

## 2. Results

### 2.1. Total Flavonoid Content, Total Polyphenol Content, Total Ascorbic Acid Content and Antioxidant Capacity in C. Aurantium By-Product Extracts

Regarding TFC, as shown in Table 1, no statistically significant differences (*p* > 0.05) were observed among experiments #1 and the four center points (experiments #5 to #8), with all these treatments exhibiting the highest TFC values, ranging from 1474.95 to 1493.97 mg QE/100 g DM. In contrast, the lowest TFC values were obtained for the axial point with the highest molar ratio (Exp #10; MR: 1:2.20 mol/mol and AW: 50%) and the axial point with the highest water content (Exp #12; MR: 1:1.5 mol/mol and AW: 64.14%), with values of 1086.33 ± 30.1 and 1079.96 ± 30.1 mg QE/100 g DM, respectively.

Concerning TPC, the highest polyphenol content was obtained in Exp #11 (MR: 1:1.5 mol/mol and AW: 35.85%), reaching 4276.94 ± 25.4 mg GAE/100 g DM, which was significantly higher (*p* < 0.05) than the remaining treatments (Table 1). Conversely, the lowest TPC value corresponded to Exp #12 (MR: 1:1.5 mol/mol and AW: 64.14%), with 2556.71 ± 42.3 mg GAE/100 g DM.

Regarding TAA, Exp #2 (MR: 1:2 mol/mol and AW: 40%) exhibited the highest ascorbic acid content (*p* < 0.05), with 2077.66 ± 21.3 mg/100 g DM (Table 1). On the other hand, the lowest TAA content was observed in Exp #12 (MR: 1:1.5 mol/mol and AW: 64.14%), which yielded 1369.18 ± 13.2 mg/100 g DM.

Antioxidant capacity results showed that Exp #1 (88.09 ± 0.1% inhibition), Exp #2 (87.80 ± 0.1% inhibition) and Exp #6 (87.45 ± 0.1% inhibition) were the highest inhibition percentages, ranging from 87.45 to 88.09% inhibition (*p* > 0.05) (Table 1). However, Ax decreased noticeably in axial experiments, particularly in and Exp #12 (75.86 ± 0.7% inhibition), where the lowest antioxidant capacity was achieved.

### 2.2. Optimization of Total Flavonoid Content by Response Surface Methodology

The results obtained from the initial experimental design, comprising the first eight experimental runs, were subjected to first-order regression analysis to evaluate the fitting of the response variables to a linear model. The corresponding *p*-values and coefficients of determination (R^2^) for TFC, TPC, TAA, and Ax are summarized in Table 2.

Among the evaluated responses, only TFC exhibited a lack of fit to the first-order model, indicating that the experimental design could be extended by incorporating the four axial (star) points according to RSM. The analysis of the complete experimental design, comprising the 12 experimental runs (first eight experiments plus four axial points), showed that TFC fitted a second-order model (R^2^ = 81.24%; *p* = 0.0001). The regression coefficients obtained from this model were used to establish the predictive equation, Equation (1), describing the effects of MR and AW on TFC.

(1) $$\mathrm{TFC}=-2340.12\;+\;1143.63\;X_{1}\;+\;131.244\;X_{2}\;-\;483.03\;X_{1}^{2}\;-\;1.42519\;X_{2}^{2}\;+\;3.08929\;X_{1}X_{2}$$

TFC = Total Flavonoid Content (expressed as mg QE/100 g DM).

$X_{1}$ = Molar ratio of fructose per 1 mol of choline chloride (MR).

$X_{2}$ = Percentage of water added to NADES (AW).

**Figure 1:**
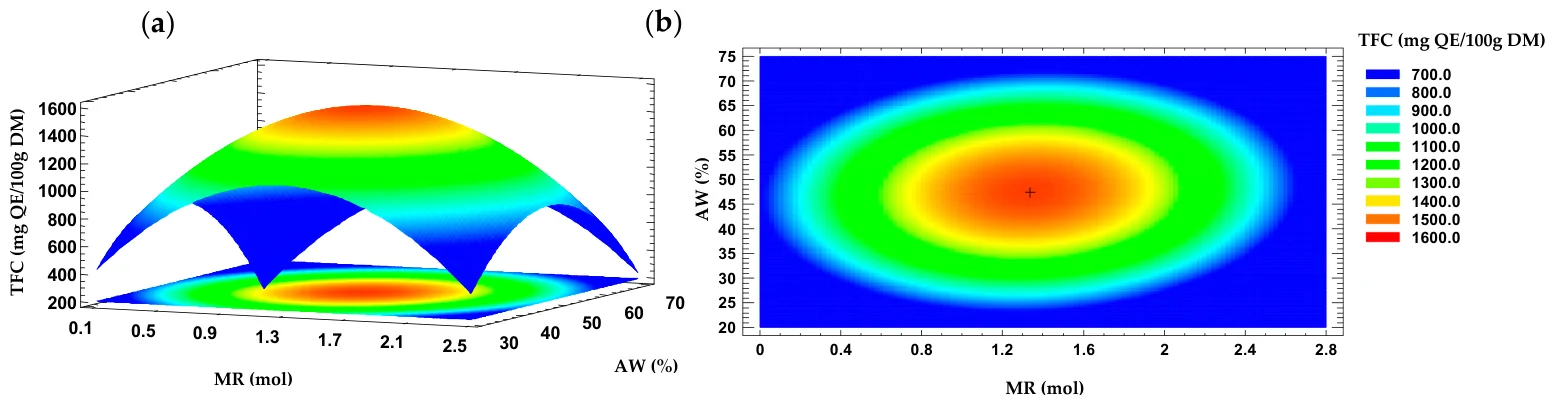
(**a**) Response surface and (**b**) contour plots for total flavonoid content as a function of the molar ratio of fructose to choline chloride and added water. The “+” symbol indicates the predicted optimal conditions. TFC = total flavonoid content; MR = molar ratio of fructose per 1 mol of choline chloride; AW = percentage of added water to NADES; QE = quercetin equivalents; DM = dry mass.

According to the mathematical model, the optimal conditions predicted to maximize total flavonoid content corresponded to a MR of 1:1.33 (choline chloride/fructose, mol/mol) and an AW of 47.48%, yielding a predicted maximum TFC value of 1540.16 mg QE/100 g DM. The response surface (Figure 1a) exhibited a maximum plateau region, where the red-colored area represents the highest predicted total flavonoid content and the blue-colored region corresponds to the lowest response. Similarly, the contour plot (Figure 1b) illustrates the distribution of TFC as a function of the evaluated factors, with the cross symbol (+) indicating the maximum predicted TFC from the optimum predicted conditions by the mathematical model.

#### Model Validation for Total Flavonoid Content

To validate the predictive model obtained through RSM, the optimal extraction conditions predicted by the mathematical model (MR = 1:1.33 mol/mol and AW = 47.48%) were experimentally evaluated.

Under these conditions, the model predicted TFC of 1540.16 mg QE/100 g DM, whereas the experimental validation yielded a value of 1576.63 ± 4.9 mg QE/100 g DM. The relative error between the predicted and experimental values was 2.37%, indicating good agreement between the mathematical model and the experimental results. Therefore, the developed model was considered suitable for predicting TFC under the evaluated extraction conditions. The optimal extract was analyzed through UPLC, and the main compounds are shown in next section, while a chromatogram of the optimal extract is shown in the Supplementary Materials (Figure S1).

### 2.3. Evaluation of the Profile of Phenolic Compounds in C. Aurantium By-Product Extracts

**Figure 2:**
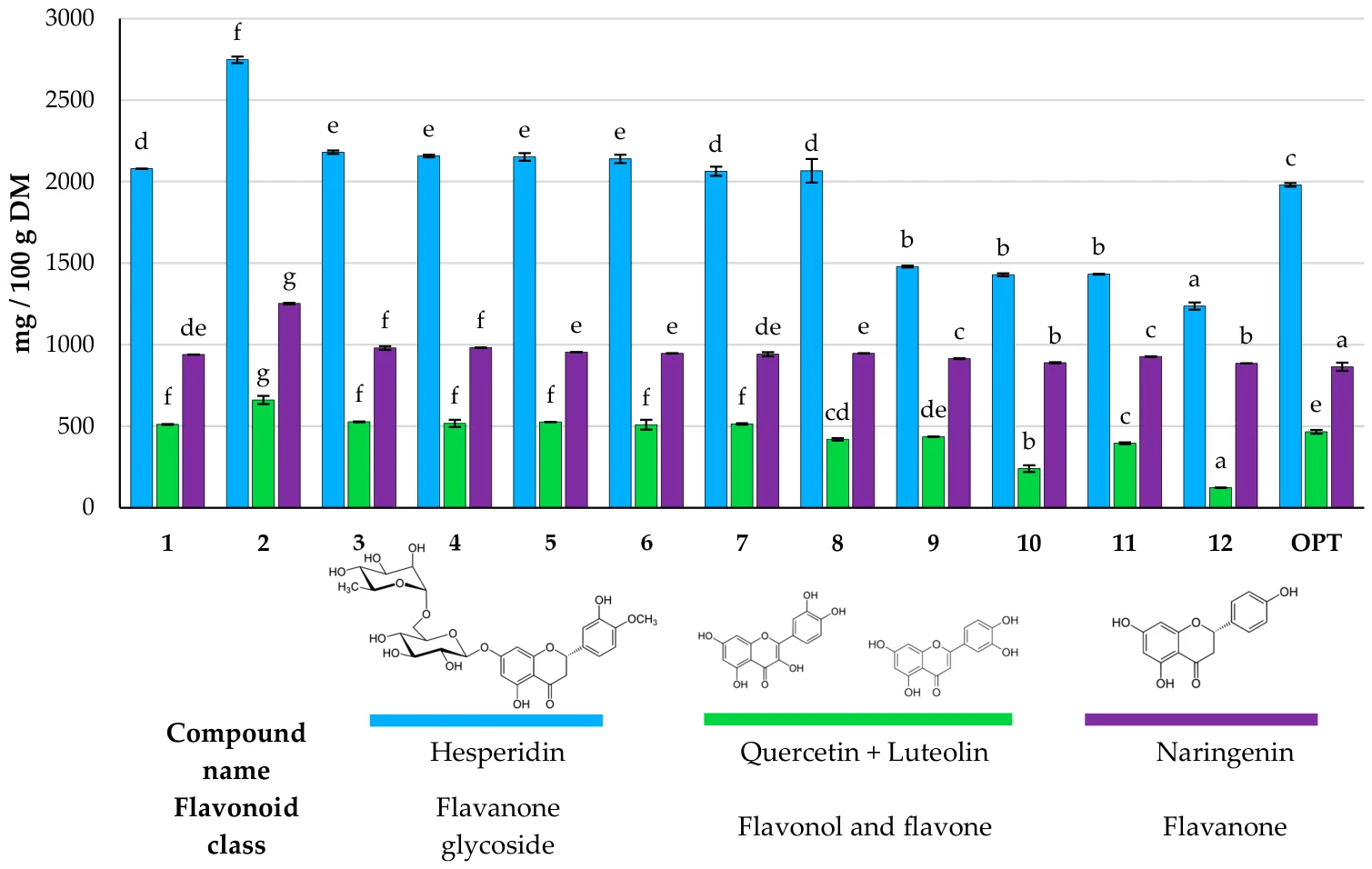
Major individual flavonoids in *C. aurantium* by-product extracts obtained using choline chloride-based NADESs with fructose as the HBD. Different letters on bars of the same color indicate statistically significant differences among individual flavonoids (LSD, *p* < 0.05). Numbers represent the different extraction conditions across the 12 experiments (Table 1). OPT: Optimal NADES system extract.

From all the flavonoids identified, hesperidin, quercetin + luteolin and naringenin were the predominant compounds in the *C. aurantium* by-product extracts. The highest concentrations of all three flavonoids were obtained in Exp #2 (MR: 2 mol of fructose per mol of choline chloride and AW: 40%), reaching values of 2747.1 ± 19.9 mg/100 g DM, 660.6 ± 25.1 mg/100 g DM and 1251.9 ± 4.91 mg/100 g DM, respectively (Figure 2). The concentrations of the remaining identified phenolic compounds in all CCD treatments are presented in Table S1 (Supplementary Materials).

Additionally, first-order regression analyses were performed for the individual phenolic compounds identified to evaluate whether any of them exhibited a lack of fit that would justify further optimization through RSM. However, none of the evaluated compounds showed a significant lack of fit (*p* > 0.05). Therefore, no additional optimization was carried out for individual phenolic compounds, and the phenolic profile was used only to characterize the extracts obtained under the different extraction conditions.

### 2.4. Physochemical Characterization of Optimal NADES System

To gain further insight into the physicochemical properties associated with the extraction performance of the optimized NADES, the solvent system was characterized by determining its spectroscopic profile using FTIR and Raman spectroscopy, viscosity, density, and pH. These analyses provided information on the structural and physicochemical features that may influence the extraction efficiency of flavonoids.

#### 2.4.1. FTIR and Raman Characterization of Optimal NADES System

**Figure 3:**
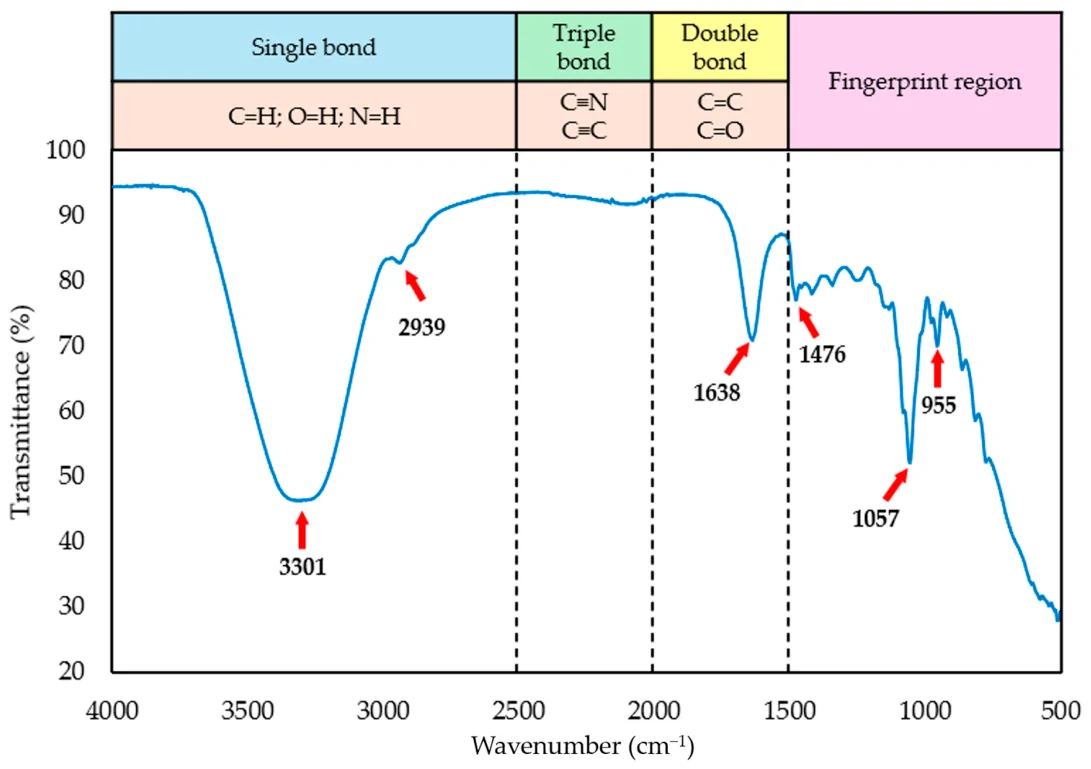
Fourier-transform infrared spectrum of NADES composed of choline chloride and fructose.

The FTIR spectrum of the optimal NADES is presented in Figure 3. The broad absorption band centered at 3301 cm^−1^ corresponds to the O-H stretching vibrations associated with the hydroxyl-containing components of the system. The band observed at 2939 cm^−1^ is attributed to the C-H stretching vibrations of the organic components of the solvent [23].

The FTIR spectrum of the ChCl-Fructose NADES is expected to combine signals from both precursors with subtle modifications. For example, hydrogen bond formation in NADES can be followed by FTIR band shift or peak broadening in either NH/OH or C=O region mainly. It has been reported in the literature that ChCl shows bands at 3218 cm^−1^ and 3017 cm^−1^ while fructose exhibits a peak at 3340 cm^−1^. In the obtained spectra of the ChCl-Fructose NADES, a single broad band peaking at 3301 cm^−1^ was observed, suggesting a red shift and therefore the presence of hydrogen bonds. In the prepared NADES, an intense band was observed in the carbonyl region at 1638 cm^−1^, which may be assigned to bending vibration of adsorbed water molecules, hydrogen bond interactions or even NH bending. In addition, the peak at 1476 cm^−1^ corresponds to CH_2_/CH_3_ deformation vibrations associated with the cationic nitrogen (quaternary ammonium cation) mainly related to choline chloride. In the fingerprint region of the eutectic solvent, the absorption band at 955 cm^−1^ is characteristic of C-O and C-C vibrational modes within the carbohydrate structure of fructose combined with choline chloride. Nevertheless, as spectra of the individual components were not obtained under the same experimental conditions, the observed spectral changes cannot be considered conclusive evidence of the formation of a new supramolecular NADES structure.

**Figure 4:**
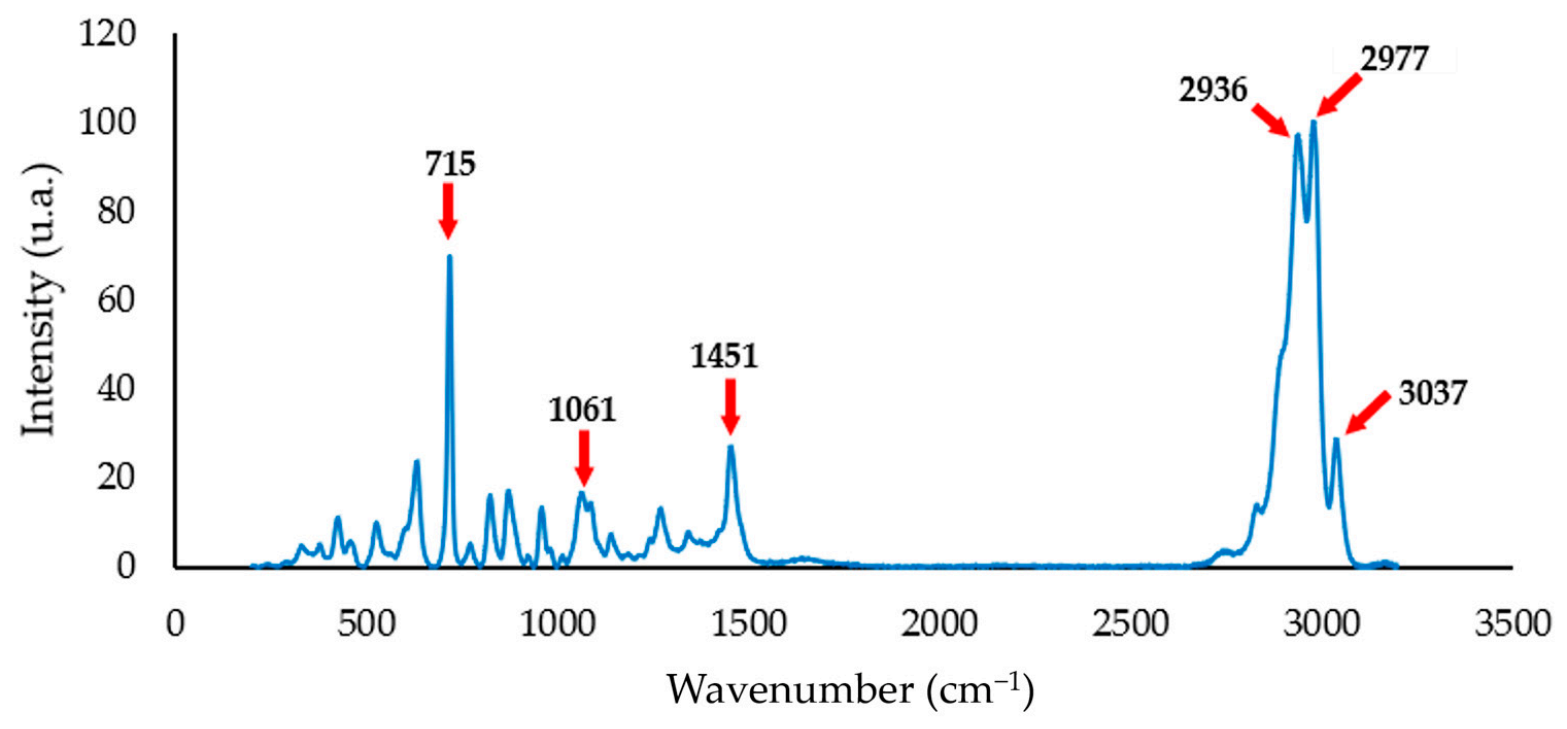
Raman spectrum of NADES composed of choline chloride and fructose.

Raman spectroscopy is a complementary vibrational technique to FTIR, providing additional structural information on molecular vibrations while being less sensitive to the presence of water. Therefore, Raman analysis was performed to further characterize the optimal NADES, and the resulting spectrum is presented in Figure 4.

The band observed at 715 cm^−1^ is associated with choline chloride (C-N) and skeletal vibrations involving C-C and C-O bonds characteristic of the carbohydrate structure. An additional band at 1061 cm^−1^ was assigned to C-O and C-C stretching vibrations, which are typical of glycosidic and alcohol groups present in fructose. The signal located at 1451 cm^−1^ corresponds to CH deformation vibrations mainly attributed to the methyl and methylene groups of choline chloride or even OH bending or CH_2_OH scissoring in fructose. In the high-wavenumber region, the intense bands at 2936, 2977, and 3037 cm^−1^ were assigned to C-H stretching vibrations of CH_2_, CH_3_ and CH groups belonging to the NADES constituents. Overall, the Raman spectrum shows characteristic vibrational features associated with the components of the choline chloride–fructose system [24].

#### 2.4.2. Viscosity, Density and pH of Optimal NADES System

**Figure 5:**
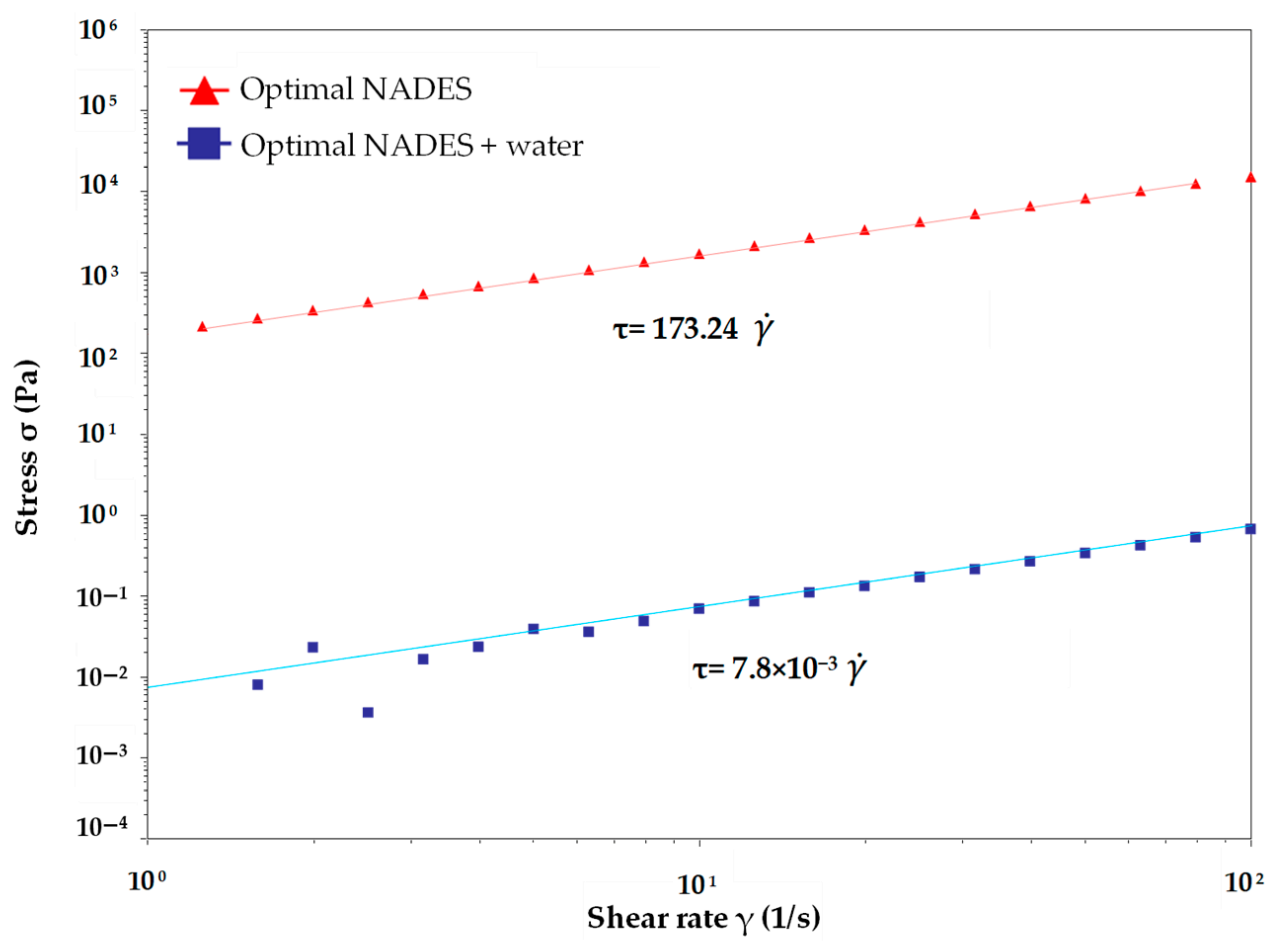
Viscosity as a function of shear rate for the optimal NADES system. The red line represents the optimal NADES prior to water addition, whereas the blue line corresponds to the optimal NADES after water addition (47.48%). The equations shown on the graph correspond to the Newtonian model ($\tau =\mu \dot{\gamma }$), where τ is the shear stress, μ is the viscosity, and $\dot{\gamma }$ is the shear rate.

The flow curves of the optimal NADES before and after water addition are presented in Figure 5. In both cases, a linear relationship between shear stress and shear rate was observed, indicating Newtonian flow behavior throughout the evaluated range. However, the addition of water markedly reduced the viscosity of the solvent, decreasing it from 173.24 ± 6.56 to 7.8 × 10^−3^ ± 0.0002 Pa·s (*p* < 0.05). This reduction is attributed to the weakening of the hydrogen bond network within the eutectic system, which increases molecular mobility and facilitates solvent flow. Such behavior is advantageous for extraction processes because lower viscosity enhances mass transfer between the solvent and the plant matrix, thereby improving the recovery of target flavonoids [25,26].

The physicochemical characterization of the optimal NADES was complemented by the determination of its density and pH, which provided additional information regarding the physical properties of the solvent system. The optimal NADES exhibited a density of 1.17 ± 0.4 g mL^−1^ and a pH value of 3.97 ± 0.06. Together with the FTIR, Raman, and viscosity analyses, these parameters contribute to the overall characterization of the optimized solvent, providing a comprehensive description of its physicochemical properties.

## 3. Discussion

The total flavonoid content obtained in the present study (1576.63 ± 4.9 mg QE/100 g DM) was higher than that reported for conventional extraction methods applied to *C. aurantium*. Mansour et al. [27] evaluated methanolic extracts obtained from different fruit fractions (albedo, flavedo, and pomace) at different maturity stages and reported a maximum TFC of 899 ± 10.9 mg QE/100 g DM in the albedo of unripe fruits. Likewise, Zeghad et al. [28] reported a maximum TFC of 727 ± 4 mg QE/100 g DM using a combination of conventional maceration and ultrasound-assisted extraction with a methanol/water (70:30, *v*/*v*) mixture. The higher flavonoid recovery achieved in the present study may be attributed to the synergistic effect between the optimized NADES composition and ultrasound-assisted extraction, which enhances solvent penetration and mass transfer while improving the solubilization of flavonoids through extensive hydrogen bond interactions [29]. Nevertheless, since conventional solvents were not evaluated under the same extraction conditions, comparisons with previous studies should be interpreted as contextual rather than as direct evidence of relative extraction efficiency.

The optimized TFC obtained in this study was lower than the values reported for optimized ultrasound-assisted extraction using organic solvents. Abdallah et al. [30] for example, optimized the extraction process from *C. aurantium* fruit peel using 80% acetone, a solid-to-liquid ratio of 0.024 g/mL, and a sonication time of 34.7 min, obtaining 7550 ± 559 mg naringin equivalents/100 g DM. The lower flavonoid content obtained in the present study may be attributed to differences in both the extraction conditions and the plant matrix. Abdallah et al. [30] employed a considerably lower solid-to-liquid ratio (0.0224 g/mL), whereas the ratio used in the present study was 1 g/12 mL (0.083 g/mL). A lower solid-to-liquid ratio may improve the interaction between the solvent and the plant matrix by increasing solvent availability and reducing local saturation during extraction. Also, the raw material used in the present study was a mixture of different parts of *C. auranium* fruit after essential oil and juice process extraction, and according to Alizadeh et al. [31], essential oils may contain phenolic compounds. However, despite their higher extraction efficiency, organic solvents present important limitations related to toxicity, solvent recovery, and environmental impact [32]. In contrast, NADESs are composed of naturally occurring metabolites and can often be used directly in subsequent applications without requiring solvent removal or purification steps, making them a more sustainable alternative for the recovery of bioactive compounds [33].

The TFC obtained in the present study was also lower than that reported by Liu et al. [34], who optimized the ultrasound-assisted extraction of flavonoids from Aurantii Fructus Immaturus (the dried immature fruit of *C. aurantium* L.) using a betaine–urea DES. Under the optimal extraction conditions (molar ratio 1:3 HB:HBD mol/mol, 30% water, liquid-to-solid ratio of 30 mL/g, 50 °C, and 50 min), these authors achieved a flavonoid content of 16,816 ± 47 mg/100 g DM. Differences between both studies may be associated with the solvent composition, extraction conditions and, importantly, the plant matrix employed, since Aurantii Fructus Immaturus and the industrial by-products of *C. aurantium* differ considerably in their phytochemical composition and flavonoid distribution.

Although TPC, TAA and Ax were not selected as response variables for optimization, all three exhibited significant values under the evaluated extraction conditions. The highest TPC reached 4276.94 ± 25.4 mg GAE/100 g DM (axial point; MR: 1:1.5 mol/mol and AW: 35.85%), exceeding the value reported by Abdallah et al. [30], who obtained 3376 ± 563 mg GAE/100 g DM using ultrasound-assisted extraction optimized with 80% acetone, although it is lower than the 9584 mg GAE/100 g DM reported by Hao et al. [35] following a Box–Behnken optimization with aqueous ethanol (extraction solvent 70.31% ethanol, extraction time 51.73 min, extraction temperature 61.94 °C, and liquid-to-solid ratio 35.63 mL/g) from *C. aurantium* blossoms. Likewise, the highest TAA value (2077.66 ± 21.3 mg/100 g DM) was obtained in Exp #2 (MR: 1:2 mol/mol and AW: 40%), slightly exceeding the maximum value previously reported by our research group (1964.9 ± 33.7 mg/100 g DM) (*p* < 0.05) using the same HBA/HBD and MR combination but under different extraction conditions, including a higher water content (50% in the previous study vs. 40% now) and an ultrasonic bath instead of an ultrasonic probe [12]. According to Islam et al. [36] ultrasonic probe (direct application) is more efficient than ultrasound bath (indirect application) for the recovery of bioactive compounds, due ultrasonic energy is intensified in a particular zone creating a higher cavitation effect, resulting in a lower extraction time. It should be considered that, due to the spectrophotometric nature of the method and the chemical complexity of the plant matrix, potential contributions from other UV-absorbing compounds to the measured absorbance cannot be completely excluded. Therefore, the TAA results should be interpreted within the analytical constraints of the method. Finally, the maximum antioxidant capacity (88.09 ± 0.1% inhibition) was slightly lower than the 100% DPPH inhibition reported by Ramírez-Sucre et al. [37] for fresh *C. aurantium* peel, which may be explained by differences in the raw material, since the industrial by-products used in the present study had previously undergone juice and essential oil extraction, processes that may promote the partial degradation of antioxidant compounds. These findings also suggest that each of these responses could potentially be further enhanced through dedicated optimization strategies, since the extraction conditions that maximized TFC were not necessarily those yielding the highest TPC, TAA, or Ax values.

Regarding individual phenolic compounds, hesperidin was the predominant flavonoid identified in the extracts, followed by naringenin and quercetin + luteolin. This profile is consistent with the characteristic phenolic composition of *C. aurantium*, in which hesperidin and naringenin have been widely reported as the major flavonoids naturally accumulated in citrus tissues, particularly in the peel [38].

The highest concentrations of hesperidin (2474.1 ± 19.9 mg/100 g DM) and naringenin (1251 ± 4.91 mg/100 g DM) were obtained under the extraction conditions of Exp #2 (MR: 2 mol of fructose per mol of choline chloride and AW: 40%). Liu et al. [34] optimized the extraction of flavonoids from *C. aurantium* peel using a betaine/ethanediol (1:4 mol/mol) DES containing 40% water, a solid-to-liquid ratio of 1:100 g/mL, and heating extraction at 60 °C for 30 min, obtaining 2872 ± 0.35 mg/100 g DM of hesperidin and 3835 mg/100 g DM of naringin. Although the hesperidin content was comparable to that obtained in the present study, considerably higher concentrations of naringenin were reported under their optimized extraction conditions.

For quercetin + luteolin, the maximum concentration obtained in the present study (660.65 ± 25.1 mg/100 g DM) was markedly higher than that reported by Ramírez-Sucre et al. [37], who extracted 160.99 ± 1.75 mg/100 g DM from *C. aurantium* peel using a glucose-based NADES (choline chloride/glucose, 1:2 mol/mol) containing 70% water and ultrasound-assisted extraction.

The FTIR and Raman analyses showed characteristic vibrational features of the optimal NADES and provided complementary spectroscopic information on its chemical composition. In the FTIR spectrum, the broad and intense absorption band observed at 3301 cm^−1^ is attributed to O-H stretching vibrations and is consistent with hydrogen bond interactions commonly reported in NADESs [23]. The broad character of this band may be associated with hydrogen bond interactions and with the contribution of water to the O-H stretching region. In agreement with the present results, Delgado-Mellado et al. [39] reported that increasing the water content in choline chloride-citric acid DES up to 40% enhanced the intensity of the O-H region, suggesting that water actively participates in the reorganization of the hydrogen bond network rather than simply acting as a diluent. Similarly, Sakurai et al. [26] associated broad O-H absorption with the supramolecular hydrogen bond interactions involved in DES formation. Additional bands observed at 1476 cm^−1^ and within the 1053–1057 cm^−1^ region is consistent with the C–H/N–CH_3_ bending vibrations of choline chloride and the C-O/C-N stretching vibrations reported for choline chloride-based eutectic systems [23].

Raman spectroscopy complemented the FTIR analysis by providing additional information on the molecular structure of the optimal NADES while being less affected by the presence of water. The Raman spectrum showed characteristic vibrational bands associated with the components of the choline chloride–fructose system. Similar observations were reported by Sakurai et al. [26], who demonstrated that water contents between 5 and 40% do not produce significant changes in the vibrational behavior of DESs, indicating that moderate water addition preserves the molecular structure of the eutectic solvent while reducing viscosity.

The marked decrease in viscosity observed after water addition demonstrates the strong influence of water on the physicochemical properties of the optimized NADES. The dynamic viscosity decreased from 173.24 ± 6.56 Pa·s in the pure solvent to 7.8 × 10^−3^ ± 0.0002 Pa·s after the addition of 47.48% water, corresponding to an approximately 22,200-fold reduction, equivalent to 4.35 orders of magnitude (the viscosity of water is 1.0 × 10^−3^ Pa·s). This pronounced reduction may be associated with the partial disruption of the supramolecular hydrogen bond network established between the HBA and HBD. Water molecules may compete for hydrogen bond interactions, increasing the free volume of the system, enhancing molecular mobility, and consequently reducing resistance to flow. The addition of water to NADES can reduce viscosity by weakening intermolecular interactions; however, its effect on the supramolecular structure depends on the system and water concentration [40]. Dai et al. [41] reported complete disruption of hydrogen bond interactions in some NADESs upon addition of 50% (*v*/*v*) water, whereas other studies [42] have reported the preservation of the molecular-level NADES structure at water contents of 50% or below. Therefore, water content should be carefully controlled, and a universal concentration threshold for preservation of the NADES structure cannot be assumed. At sufficiently high water contents, progressive restructuring may lead to a transition from a “water in NADES” system toward a “NADES in water” system, in which water increasingly governs the molecular environment [43]. Considering the relatively high water content of the optimized formulation (47.48%), the pronounced decrease in viscosity observed in the present study may indicate extensive hydration and restructuring of the NADES, although the occurrence of such a transition cannot be conclusively established from the present results. This behavior is consistent with the hygroscopic nature of most NADESs, in which water acts as a plasticizing agent, weakening the interactions between the original components and generating cavities that facilitate molecular movement, often leading to viscosity reductions of one or more orders of magnitude [44,45]. Notably, 47.48% AW was identified by the optimization model as the condition that maximized TFC recovery within the evaluated experimental domain. Therefore, the substantial reduction in viscosity and the associated increase in molecular mobility at the optimized water content could favor solvent penetration and mass transfer within the plant matrix, potentially contributing to the observed extraction performance. Although the pronounced viscosity decrease is consistent with substantial changes in intermolecular interactions upon hydration, viscosity alone does not allow the extent of structural reorganization of the system to be conclusively established. The optimal NADES exhibited a considerably higher viscosity than that reported by Gajardo-Parra et al. [44] for a choline chloride:1,4-butanediol NADES (1:3 HBA/HBD molar ratio), which showed a dynamic viscosity of 0.0874 Pa·s at 25 °C. This difference can be attributed to the nature of the hydrogen bond donor, as sugar-based NADESs generally exhibit higher viscosities than polyol-based systems due to the formation of a more extensive hydrogen bond network. In addition, the molar ratio between the components also contributes to the rheological behavior of NADES.

The optimal NADES exhibited a density of 1.17 ± 0.04 g mL^−1^ at 25 °C, which is comparable to the density reported by Rao et al. [45] for a choline chloride/urea 1:2 (HBAmol/HBD mol) deep eutectic solvent (DES) (1.1932 g mL^−1^ at 29.85 °C). Likewise, the pH of the optimal NADES (3.97 ± 0.06) was close to the values reported by Liu et al. [34] for sugar and polyol-based DESs, which exhibited weak acidity with pH values ranging from 4.6 to 6.4. Slight differences may be attributed to variations in the hydrogen bond donor, molar ratio, and water content employed in the solvent formulation. The pH of DESs is primarily governed by the acid and alkaline properties of the HBA and HBD and can be modified by changes in the solvent composition, including the nature of the HBD and the MR between the components. Previous studies have shown that increasing the MR of fructose in choline chloride-based DESs results in higher pH values. In addition to being a physicochemical characteristic of the solvent, pH has been recognized as an important factor influencing extraction performance, as it affects the interaction between the DES and the target compounds and may facilitate plant cell wall disruption, thereby promoting the release of bioactive molecules. Therefore, the pH of the optimal NADES may have contributed, together with other physicochemical properties of the solvent, to its extraction capacity [19,46].

## 4. Materials and Methods

### 4.1. Raw Material

Fresh *C. aurantium* by-products were collected from ARPEN Juicer S.P.R. de R.L. de C.V., located in Akil, Yucatán, Mexico (20°17′14.7″ N, 89°23′0.9″ W), immediately after the extraction of juice and essential oils. The by-products consisted of a mixture of albedo, flavedo, pulp, and seeds.

### 4.2. Drying Process of Citrus Aurantium By-Product

A drying process was carried out following the method described by Mendoza-Osorno et al. [47] with some modifications. Fresh by-products were first frozen at −20 °C for a minimum of 48 h and subsequently freeze-dried (FreeZone, LABCONCO^®^, Kansas City, MO, USA) at −80 °C under a pressure of 0.005 mBar for 96 h, until a final moisture content of approximately 3% was reached. The dried material was then ground using a grinder (Hukën × MasterChef^®^, Naucalpan de Juárez, Estado de México, México) and sieved through a #35 mesh (500 µm; Fisher Scientific, Boston, MA, USA). Finally, the resulting powder was stored in a desiccator, protected from light and maintained at room temperature (30–32 °C) until the extraction experiments.

### 4.3. Experimental Design

Response surface methodology (RSM) was employed to optimize the extraction conditions. A 2^2^ Central Composite Design (CCD) with four center points and four axial (star) points was employed. The experimental matrix and the corresponding coded and actual values of the independent variables are summarized in Table 3.

The choline chloride–fructose system evaluated in the present study was selected based on a previous experimental screening conducted by our research group, in which fructose, glucose, and glycerol were evaluated as HBDs in choline chloride-based NADESs. Fructose-based systems exhibited the highest TPC and antioxidant capacity among the evaluated formulations and were therefore selected for further investigation [12]. The experimental design consisted of two independent variables evaluated at two levels, molar ratio (MR) of fructose per 1 mol of choline chloride (1:1 mol/mol, 1:2, mol/mol) and the percentage of added water (AW) (40%, 60%), together with four center points and four axial points, resulting in 12 experimental runs. The center points were established at a choline chloride/fructose molar ratio of 1:1.5 (mol/mol) and 50% AW. According to the CCD, the axial points for the MR factor corresponded to 1:0.79 and 1:2.20 (mol/mol), both combined with the center AW value (50%), whereas the axial points for the AW factor were established at 35.85% and 64.14%, using the center MR value (1:1.5 mol/mol). The response variable used for optimization was total flavonoid content (TFC); however, total phenolic content (TPC), total ascorbic acid (TAA), antioxidant capacity (Ax), and the phenolic profile were also determined for all experimental treatments.

### 4.4. Preparation of NADES

NADESs were prepared following the heating and stirring method described by Mansinhos et al. [48]. Choline chloride (molecular weight: 139.63 g/mol; Tm = 302 °C) was used as the hydrogen bond acceptor (HBA), whereas fructose (molecular weight: 180.15 g/mol; Tm = 103 °C) served as the hydrogen bond donor (HBD). Both components were mixed at molar ratios of 1:1 (−1, low level), 1:1.5 (0, center point), and 1:2 (1, high level), where the HBD ratio was the one that changed. The mixtures were heated at 90 °C with continuous stirring for 2 h until transparent liquids were obtained. Finally, water was added at 40 (−1, low level), 50 (0, center point), and 60% (1, high level) (*w*/*w*) of the total mixture weight to reduce the viscosity of the NADESs (Table 3).

### 4.5. Ultrasound Assited Extraction of Bioactive Compounds Using NADESs

Ultrasound-assisted extraction was carried out following the methodology described by Fernández-Cabal et al. [12], with slight modifications. Briefly, 2 g of sample was combined with 24 mL of the corresponding NADES, according to the experimental design, and homogenized using a vortex mixer. The mixtures were then treated with an ultrasonic probe (Sonics Vibra-Cell^®^, model CV505, Sonics^®^, New York, NY, USA) at 750 W, 20 kHz, and 30% amplitude for an effective sonication time of 5 min. To minimize the temperature increase generated during probe sonication, the samples were maintained in a cold-water bath throughout the extraction, and the temperature was kept below 20 °C. Following extraction, the samples were subjected to a two-step centrifugation procedure using a refrigerated benchtop centrifuge (Hettich^®^ Mikro 22R, Beverly, MA USA): initially at 4700 rpm for 30 min at 4 °C, followed by three consecutive centrifugation cycles of the recovered supernatant at 15,000 rpm for 30 min at 4 °C. The final supernatants were filtered through 0.22 μm nylon membrane filters (Whatman^®^ UNIFLO^®^ 25, 9910-2502, Cytiva, Marlborough, MA, USA) and transferred to amber chromatographic vials and stored under refrigeration (<18 °C) until further analysis.

### 4.6. Spectrofotometric Assays

#### 4.6.1. Total Flavonoid Content Determination

Total flavonoid content (TFC) was determined according to the colorimetric method described by Estrada-Sierra et al. [49]. Before analysis, each extract was diluted 1:10 with distilled water. For the assay, 100 μL of the diluted extract was mixed with 250 μL of distilled water and 75 μL of 5% NaNO_2_. After 6 min of incubation, 150 μL of 10% AlCl_3_ was added, and the mixture was allowed to react for an additional 5 min. Subsequently, 500 μL of 1 M NaOH and 425 μL of distilled water were incorporated, followed by mixing and incubation in the dark for 30 min. Absorbance was then recorded at 510 nm using a UV–Vis spectrophotometer (Genesys 140, Thermo Scientific^®^) using distilled water as blank. Quantification was performed using a quercetin calibration curve ranging from 100 to 1400 μg/mL (R^2^ = 0.9993), and the results were expressed as milligrams of quercetin equivalents (QE) per 100 g of dry mass (DM).

#### 4.6.2. Total Polyphenol Content Determination

The total polyphenol content (TPC) of the extracts was determined according to the Folin–Ciocalteu method described by Singleton et al. [50], with slight modifications. Prior to analysis, each extract was diluted 1:50 with distilled water. For the assay, 1 mL of the diluted extract was mixed with 3 mL of distilled water and 250 μL of Folin–Ciocalteu reagent (1:2, *v*/*v*). After allowing the reaction to proceed for 5 min, 750 μL of 20% Na_2_CO_3_ and 950 μL of distilled water were added. The reaction mixtures were vortexed and incubated in the dark for 30 min before measuring the absorbance at 765 nm using a UV–Vis spectrophotometer (Genesys 140, Thermo Scientific^®^, Mexico City, Mexico) using distilled water as blank. Quantification was performed using a gallic acid calibration curve (5–100 μg/mL; R^2^ = 0.9994) and the results were expressed as milligrams of gallic acid equivalents (GAE) per 100 g of DM.

#### 4.6.3. Total Ascorbic Acid Determination

Total ascorbic acid content (TAA) was determined following the method reported by Hagos et al. [51], with slight modifications. Prior to analysis, each extract was diluted 1:50 using a water/acetonitrile mixture (80:20, *v*/*v*). Absorbance was measured at 244 nm with a UV–Vis spectrophotometer (Genesys 140, Thermo Scientific^®^) using a water/acetonitrile mixture as blank. Quantification was carried out using a five-point calibration curve prepared with standard ascorbic acid solutions ranging from 5 to 50 μg/mL (R^2^ = 0.9997). The results were expressed as mg per 100 g of DM.

#### 4.6.4. Antioxidant Capacity Assessment

Antioxidant capacity (Ax) was determined using the 2,2-diphenyl-1-picrylhydrazyl (DPPH) radical scavenging assay according to the method described by Chel-Guerrero et al. [52]. A DPPH solution was prepared by dissolving 3.3 mg of DPPH in methanol to a final volume of 100 mL, and its absorbance was adjusted to 0.700 ± 0.002 at 515 nm. Subsequently, 100 μL of each extract was mixed with 3.9 mL of the adjusted DPPH solution. After vortexing, the reaction mixtures were incubated in the dark for 30 min, and the absorbance was measured at 515 nm using a UV–Vis spectrophotometer (Genesys 140, Thermo Scientific^®^) using methanol as blank. The antioxidant capacity was expressed as the percentage of DPPH inhibition, calculated according to Equation (2).

(2) $$\%\;DPPH\;inhibition=\left(\frac{A\;control-A\;sample}{A\;control}\right)\times 100$$

where A control represents the absorbance of adjusted DPPH and A sample the absorbance of the DPPH solution with the sample.

### 4.7. Polyphenol Profile

The phenolic profile of *C. aurantium* by-products was determined using an Acquity UPLC H-Class system equipped with a diode array detector (DAD) (Waters Corporation, Milford, MA, USA) and an Acquity UPLC HSS C18 column (100 A°, 1.8 mm, 2.1 × 50 mm) (Waters Corporation, Wexford, Leinster, Ireland), following the method described by Avilés-Betanzos et al. [53], with slight modifications. Chromatographic separation was carried out at a flow rate of 0.5 mL min^−1^ and a column temperature of 45 °C, using an injection volume of 2 μL and a detection wavelength of 280 nm. The mobile phase consisted of water containing 0.2% acetic acid (solvent A) and acetonitrile containing 0.1% acetic acid (solvent B). The elution gradient was as follows: 0–10 min from 1% B to 30% B; 10–12 min 30% B; 12–15 min from 30% B to 1% B.

Compound identification and quantification were achieved using calibration curves prepared from sixteen analytical standards at concentrations ranging from 5 to 75 μg/mL. The standards included gallic acid, protocatechuic acid, chlorogenic acid, *p*-coumaric acid, cinnamic acid, catechin, rutin, kaempferol, quercetin, luteolin, vanillin, hesperidin, neohesperidin, naringenin, apigenin, diosmetin, and quercetin + luteolin (quantified collectively, due to their overlapping peaks during analysis).

Prior to UPLC analysis, the filtered extracts obtained as described in Section 4.5 were diluted 1:20 *v*/*v* to reduce the concentration of matrix components and filtered again to 0.22 μm nylon membrane filters to prevent column clogging.

### 4.8. Physochemical Properties of Optimal NADES

#### 4.8.1. Viscosity Determination

The viscosity of the optimal NADES formulation was determined according to the method reported by Ramírez-Sucre et al. [54] with some modifications. A controlled-stress rheometer (DHR-2, TA Instruments, New Castle, DE, USA) equipped with a parallel-plate geometry (40 mm diameter) and a gap of 1050 μm was used. Measurements were performed for both the neat NADES and the corresponding water-diluted formulations. All rheological measurements were carried out at a controlled temperature of 25 °C after thermal equilibration. Flow curves were obtained by recording the shear stress (τ, Pa) as a function of the shear rate ($\dot{\gamma }$), ranged from 1 to 100 s^−1^. The rheological behavior of the systems was described using the Newtonian model, assuming a linear relationship between shear stress (τ) and shear rate ($\dot{\gamma }$), according to Equation (3):

(3) $$\tau \;=\;\eta \;\times \;\dot{\gamma }$$

where η represents the dynamic viscosity (Pa·s). Viscosity values were expressed as the mean ± standard deviation.

#### 4.8.2. Fourier-Transform Infrared (FTIR) Spectroscopy

The infrared spectra of the optimal NADES formulations were recorded following the methodology described by Avilés-Betanzos et al. [55] with some modifications using a Nicolet iS5 (Thermo Fisher Scientific Inc., Madison, WI, USA) spectrometer equipped with an attenuated total reflectance (ATR) accessory. Spectra were collected over the wavenumber range of 4000–500 cm^−1^ using 64 background scans at a spectral resolution of 8 cm^−1^. The results were recorded in transmittance mode (%).

#### 4.8.3. Raman Spectroscopy

Raman spectra were acquired according to the methodology described by Pech-Pisté et al. [56] using a Renishaw InVia Reflex Raman spectrometer (Wotton-under-Edge, Gloucestershire, UK). Spectral acquisition was performed over the wavenumber range of 3200–200 cm^−1^ using a 633 nm excitation laser operated at 50% of its maximum power. Spectra were collected with a 50× objective and an exposure time of 10 ms.

#### 4.8.4. pH Determination

The pH of the optimal NADES was determined following the method described by Sakurai et al. [26] with some modifications. An APERA pH meter (model PC820; Apera Instruments, LLC, Columbus, OH, USA) previously calibrated with standard solutions of pH 4.0, 7.0 and 10.0 was used. Measurements were performed at a controlled temperature of 25 °C.

#### 4.8.5. Density Determination

The density of the optimal NADES formulation was determined following the methodology described by Zarghampour et al. [57], with some modifications. A 25 mL pycnometer (Glassco Laboratory Equipments Pvt. Ltd., Ambala Cantt, India) and an analytical balance (OHAUS Corporation, Parsippany, NJ, USA) were used at a controlled temperature of 25 °C. The density was calculated from the difference between the mass of the filled and empty pycnometer according to Equation (4):

(4) $$p=\;\frac{Iw-Fw}{V}$$

where p is the density (g·mL^−1^), *Iw* is the mass of the pycnometer filled with the sample (g), *Fw* is the mass of the empty pycnometer (g), and *V* is the pycnometer volume (mL).

### 4.9. Statical Analysis

First and second-order model fitting, regression coefficient estimation, canonical analysis, and all statistical analyses were performed using Statgraphics Centurion XVI software (version 16.1.03; Statgraphics Technologies Inc., The Plains, VA, USA). All experiments were conducted in a randomized order, and each determination was performed in triplicate. The results are presented as the mean ± standard deviation (SD).

## 5. Conclusions

A fructose-based NADES prepared with choline chloride as the HBA was successfully optimized through RSM combined with ultrasound-assisted extraction, maximizing the recovery of total flavonoids from *C. aurantium* by-products. Although the optimization was focused exclusively on total flavonoid content, the extracts also exhibited considerable TPC, TAA and Ax values, together with high concentrations of individual phenolic compounds. These results were comparable to and in some cases exceeded those reported in previous studies, highlighting the extraction potential of the optimized solvent across different classes of bioactive compounds and suggesting that additional optimization strategies targeting these response variables could further enhance the recovery of specific bioactive compounds. Furthermore, the physicochemical characterization of the optimal NADES through viscosity, density, pH, FTIR, and Raman analyses provided valuable insight into the physicochemical properties and vibrational features of the solvent. The spectroscopic results were consistent with hydrogen bond interactions, although they did not conclusively establish the formation or preservation of a new supramolecular NADES structure. Overall, the results demonstrate that the combination of optimized NADES and ultrasound-assisted extraction represents a promising approach for the valorization of *C. aurantium* industrial by-products as a source of bioactive compounds.

## Figures and Tables

**Table 1:** Values of total flavonoid content, total polyphenol content, total ascorbic acid and antioxidant capacity from *C. aurantium* by-products obtained from central composite design (2^2^) to optimize NADES composition.

#EXP	Encoded Values		Real Values		Response Variable			
	X_1_	X_2_	MR (mol/mol)	AW (%)	TFC (mg QE/100 g DM)	TPC (mg GAE/100 g DM)	TAA (mg/100 g DM)	Ax (% Inhibition)
1	−1	−1	1	40	1493.97 ± 8.6 ^e^	3774.11 ± 67.8 ^h^	1920.51 ± 6.2 ^h^	88.09 ± 0.1 ^g^
2	1	−1	2	40	1279.72 ± 8.6 ^d^	3284.93 ± 39.5 ^e^	2077.66 ± 21.3 ^i^	87.80 ± 0.1 ^fg^
3	−1	1	1	60	1310.90 ± 34.5 ^d^	2790.47 ± 118.8 ^b^	1819.52 ± 8.8 ^ef^	86.38 ± 0.1 ^e^
4	1	1	2	60	1158.44 ± 20.9 ^bc^	2811.60 ± 74.2 ^b^	1879.93 ± 6.9 ^g^	85.95 ± 0.1 ^de^
5	0	0	1.5	50	1493.08 ± 20.5 ^e^	3527.43 ± 36.1 ^g^	1771.96 ± 35.3 ^d^	87.24 ± 0.2 ^f^
6	0	0	1.5	50	1475.13 ± 37.4 ^e^	3331.76 ± 49.4 ^f^	1818.16 ± 21.2 ^ef^	87.45 ± 0.2 ^fg^
7	0	0	1.5	50	1474.95 ± 36.7 ^e^	3060.98 ± 42.4 ^c^	1787.50 ± 9.1 ^de^	87.27 ± 0.3 ^f^
8	0	0	1.5	50	1492.03 ± 14.1 ^e^	3095.99 ± 35.3 ^c^	1817.12 ± 17.2 ^ef^	87.16 ± 0.2 ^f^
9	−√2	0	0.79	50	1238.85 ± 12.9 ^cd^	3516.79 ± 25.4 ^g^	1530.11 ± 10.5 ^b^	85.40 ± 0.3 ^d^
10	+√2	0	2.20	50	1086.33 ± 30.1 ^ab^	3150.54 ± 11.3 ^d^	1730.93 ± 12.3 ^c^	79.93 ± 0.4 ^b^
11	0	−√2	1.5	35.85	1176.49 ± 21.5 ^bc^	4276.94 ± 25.4 ^i^	1824.83 ± 17.8 ^f^	84.01 ± 0.5 ^c^
12	0	+√2	1.5	64.14	1079.96 ± 30.1 ^a^	2556.71 ±42.3 ^a^	1369.18 ± 13.2 ^a^	75.86 ± 0.7 ^a^

Note: 2^2^ central composite design with two factors (molar ratio and added water) and four center points. #EXP = experiment number; MR = molar ratio of fructose per 1 mol of choline chloride; AW = percentage of added water to NADES; TFC = total flavonoid content; TPC = total polyphenol content; TAA = total ascorbic acid; Ax = antioxidant capacity. Values are expressed as the mean ± standard deviation of triplicate measurements (n = 3). Different superscript letters within the same column indicate statically significant differences among treatments according to the LSD test (*p* < 0.05).

**Table 2:** The fit of the first-order regression model for the evaluated response variables from CCD (2^2^).

Response	*p*-Value	R^2^
**TFC**	**0.1280**	**36.62**
TPC	0.0000	88.89
TAA	0.0295	51.38
Ax	0.0000	91.94

Note: R^2^ = Coefficient of determination; TFC = total flavonoid content; TPC = total polyphenol content; TAA = total ascorbic acid; Ax = antioxidant capacity. The *p*-values above 0.05 indicate lack of adjustment to a first-order model. Bold formatting indicates the lack of fit to the first-order model.

**Table 3:** Experimental 2^2^ central composite design used to optimize the NADES formulation for flavonoid extraction from *Citrus aurantium* by-products.

#EXP	Encoded Values		Real Values		Response Variables *
	X_1_	X_2_	MR (mol/mol)	AW (%)	
1	−1	−1	1	40	Y_1_
2	1	−1	2	40	Y_2_
3	−1	1	1	60	Y_3_
4	1	1	2	60	Y_4_
5	0	0	1.5	50	Y_5_
6	0	0	1.5	50	Y_6_
7	0	0	1.5	50	Y_7_
8	0	0	1.5	50	Y_8_
9	−√2	0	0.79	50	Y_9_
10	+√2	0	2.20	50	Y_10_
11	0	−√2	1.5	35.85	Y_11_
12	0	+√2	1.5	64.14	Y_12_

Note: #EXP = experiment number; MR = molar ratio of fructose per 1 mol of choline chloride; AW = percentage of added water to NADES; * the response variables (Y_n_) for every experiment were total polyphenol content (TPC), total flavonoid content (TFC), total ascorbic acid (TAA) and antioxidant capacity (Ax).

## Data Availability

The original contributions presented in this study are included in the article/Supplementary Materials. Further inquiries can be directed to the corresponding authors.

## Supplementary Materials

The following supporting information can be downloaded at: https://www.mdpi.com/article/10.3390/molecules31183192/s1, Figure S1: Chromatogram of the optimal extract for total flavonoid content. The numbered peaks correspond to: (1) protocatechuic acid, (2) catechin, (3) vanillin, (4) p-coumaric acid, (5) cinnamic acid, (6) rutin, (7) quercetin + luteolin, (8) naringenin, (9) hesperidin, and (10) kaempferol; Table S1. Polyphenol profile from *C. aurantium* by-products of CCD (2^2^) obtained using different NADESs and ultrasound-assisted extraction.
