# Supplementary material for: Natural Deep Eutectic Solvent-Based Extraction of Flavonoids from Citrus Aurantium L. By-Products: Process Optimization and Physicochemical Characterization of Optimal System
Source: Molecules. 2026 Sep 10;31(18):3192. doi: 10.3390/molecules31183192 (PMC13609075; doi:10.3390/molecules31183192)
Supplement: Supplementary file 1 [file molecules-31-03192-s001.zip › molecules-4513612-supplementary.pdf]

Supplementary Material

Natural Deep Eutectic Solvent-Based Extraction of Flavonoids from *Citrus aurantium* L. By-Products: Process Optimization and Physicochemical Characterization of Optimal System

Joaquín Fernández-Cabal [1], Kevin Alejandro Avilés-Betanzos [1], Manuel Octavio Ramírez-Sucre [1], Juan Valerio Cauich-Rodríguez [2*], and Ingrid Mayanin Rodríguez-Buenfil [1*]

[1] Centro de Investigación y Asistencia en Tecnología y Diseño del estado de Jalisco A.C., Subsede Sureste, Tablaje Catastral 31264, Km. 5.5 Carretera Sierra Papacal-Chuburná Puerto, Parque Científico Tecnológico de Yucatán, Mérida 97302, Yucatán México;

[2] Centro de Investigación Científica de Yucatán, Unidad de materiales, Calle 43 No. 130 x 32 y 34, Colonia Chuburná de Hidalgo, Mérida C.P.97205, Yucatán, México;

* Correspondence: irodriguez@ciatej.mx; jvcr@cicy.mx

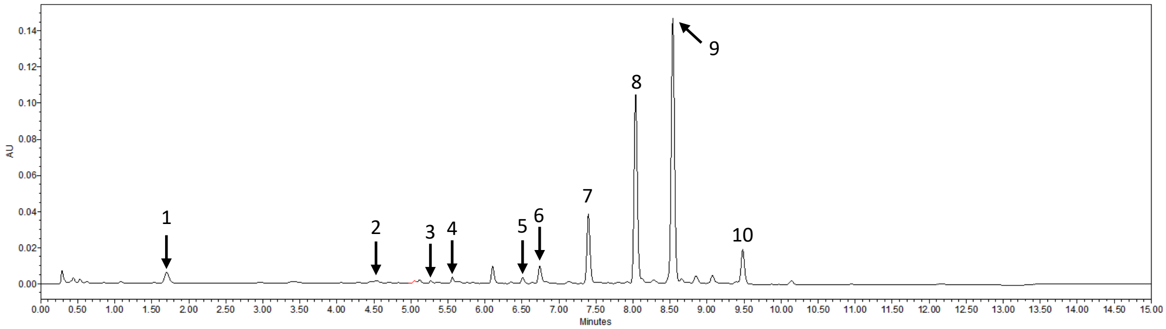

Figure S1: Chromatogram of the optimal extract for total flavonoid content. The numbered peaks correspond to: (1) protocatechuic acid, (2) catechin, (3) vanillin, (4) p-coumaric acid, (5) cinnamic acid, (6) rutin, (7) quercetin + luteolin, (8) naringenin, (9) hesperidin, and (10) kaempferol.

Table S1. Polyphenol profile from *C. aurantium* by-products of CCD (2^2) obtained using different NADESs and ultrasound-assisted extraction.

**Exp***	**Polyphenol Profile (mg / 100 g DM)**									
	**PrA**	**Ct**	**Vn**	**CoA**	**CiA**	**Rt**	**Q+L**	**Ng**	**Hp**	**Kp**
**1**	ND	37.2 ± 1.0^{a}	ND	6.4 ± 0.2^{b}	25.0 ± 0.5^{d}	115.7 ± 3.2^{d}	511.1 ± 2.7^{f}	939.3 ± 0.2^{de}	2078.8 ± 0.7^{d}	302.6 ± 8.6^{cd}
**2**	250.9 ± 3.0^{i}	38.2 ± 4.6^{a}	ND	7.1 ± 0.2^{d}	28.8 ± 0.3^{f}	138.7 ± 1.0^{g}	660.7 ± 25.1^{g}	1251.9 ± 4.9^{g}	2747.1 ± 20.0^{f}	496.6 ± 23.7^{h}
**3**	ND	34.8 ± 3.1^{a}	ND	6.4 ± 0.2^{b}	26.3 ± 0.8^{e}	128.4 ± 9.7^{f}	527.1 ± 2.2^{f}	979.4 ± 11.9^{f}	2180.8 ± 10.5^{e}	398.6 ± 7.0^{g}
**4**	57.3 ± 0.6^{e}	88.3 ± 2.0^{bc}	ND	6.4 ± 0.2^{b}	26.3 ± 0.3^{e}	125.7 ± 8.4^{ef}	517.4 ± 22.4^{f}	981.2 ± 1.5^{f}	2157.4 ± 8.1^{e}	360.5 ± 5.6^{f}
**5**	ND	98.3 ± 6.8^{cd}	ND	6.7 ± 0.3^{bcd}	24.9 ± 0.3^{d}	126.4 ± 2.7^{ef}	525.9 ± 0.8^{f}	954.2 ± 0.1^{e}	2151.7 ± 23.6^{e}	334.7 ± 0.1^{def}
**6**	9.9 ± 0.1^{c}	88.9 ± 3.1^{bc}	ND	6.6 ± 0.2^{bc}	24.5 ± 0.3^{d}	118.0 ± 1.4^{de}	509.1 ± 30.0^{f}	946.5 ± 0.1^{e}	2139.6 ± 25.6^{e}	308.8 ± 21.7^{cd}
**7**	1.6 ± 0.5^{a}	84.0 ± 8.0^{b}	ND	6.6 ± 0.2^{bc}	22.9 ± 0.2^{c}	103.5 ± 0.3^{c}	513.7 ± 4.2^{f}	941.6 ± 12.5^{de}	2063.6 ± 28.1^{d}	281.0 ± 9.1^{bc}
**8**	7.5 ± 0.8^{b}	82.8 ± 6.8^{b}	ND	6.9 ± 0.3^{cd}	22.0 ± 0.3^{b}	82.7 ± 0.2^{b}	419.6 ± 7.6^{cd}	946.8 ± 0.3^{e}	2066.4 ± 72.3^{d}	228.0 ± 12.7^{a}
**9**	25.4 ± 0.1^{d}	118.1 ± 5.9^{e}	8.7 ± 0.2^{d}	ND	ND	48.5 ± 4.6^{a}	435.8 ± 1.4^{de}	914.6 ± 2.9^{c}	1479.1 ± 5.9^{b}	248.1 ± 3.4^{ab}
**10**	98.0 ± 1.4^{h}	118.6 ± 1.7^{e}	5.6 ± 0.2^{b}	ND	ND	43.3 ± 5.3^{a}	240.0 ± 20.8^{b}	889.3 ± 3.7^{b}	1428.5 ± 9.0^{b}	345.7 ± 6.9^{ef}
**11**	84.5 ± 1.2^{g}	102.6 ± 10.8^{d}	5.6 ± 0.1^{b}	ND	ND	50.2 ± 1.2^{a}	395.3 ± 5.8^{c}	927.0 ± 1.9^{cd}	1431.8 ± 2.4^{b}	361.3 ± 2.0^{f}
**12**	82.0 ± 1.0^{f}	95.4 ± 9.1^{bcd}	6.3 ± 0.1^{c}	ND	ND	44.2 ± 0.2^{a}	122.6 ± 1.3^{a}	884.9 ± 0.3^{b}	1236.6 ± 22.2^{a}	313.4 ± 6.6^{cde}
**OPT**	82.0 ± 1.0^{f}	118.3 ± 8.4^{e}	6.3 ± 0.1^{c}	ND	ND	43.7 ± 0.2^{a}	466.0 ± 11.4^{e}	864.6 ± 24.7^{a}	1980.1 ± 11.0^{c}	363.0 ± 12.4^{fg}

Note: DM = Dry Mass; OPT = Optimum extract; PrA = Protocatechuic acid; Ct = Catechin; Vn = Vanillin; CoA = Coumaric acid; CiA = Cinnamic acid; Rt = Rutin; Q+L = Quercetin + Luteolin; Ng = Naringenin; Hp = Hesperidin; Kp = Kaempferol; ND = Not detected. Values are means ± SD (n = 3). Different superscript letters within the same column indicate statically significant differences among treatments according to the LSD test ($p < 0.05$).
